# Co-administration of dexamethasone increases severity and accelerates onset day of neutropenia in bladder cancer patients on methotrexate, vinblastine, adriamycin and cisplatin chemotherapy: a retrospective cohort study

**DOI:** 10.1186/s40780-016-0072-5

**Published:** 2017-01-11

**Authors:** Shingo Itai, Yukio Suga, Yusuke Hara, Kouji Izumi, Yuji Maeda, Yasuhide Kitagawa, Junko Ishizaki, Tsutomu Shimada, Atsushi Mizokami, Yoshimichi Sai

**Affiliations:** 1 Division of Life Sciences, Graduate School of Natural Science and Technology, Kanazawa University, Kakuma-machi, Kanazawa, 920-1192 Japan; 2 Department of Hospital Pharmacy, University Hospital, Kanazawa University, 13-1 Takara-machi, Kanazawa, 920-8641 Japan; 3 Department of Clinical Drug Informatics, Faculty of Pharmacy, Institute of Medical, Pharmaceutical and Health Sciences, Kanazawa University, Kakuma-machi, Kanazawa, 920-1192 Japan; 4 Department of Urology, University Hospital, Kanazawa University, 13-1 Takara-machi, Kanazawa, 920-8641 Japan; 5 Department of Urology, Public Central Hospital of Matto Ishikawa, 3-8 Kuramitsu, Hakusan, 924-8588 Japan; 6 Department of Clinical Pharmacokinetics, Graduate School of Medical Sciences, Kanazawa University, 13-1 Takara-machi, Kanazawa, 920-8641 Japan

**Keywords:** Cancer chemotherapy, Neutropenia, Dexamethasone, Adverse effect, Bladder cancer

## Abstract

**Background:**

Bladder cancer patients receiving methotrexate, vinblastine, adriamycin and cisplatin (MVAC) chemotherapy are co-administered with dexamethasone as an anti-emetic. We examined whether or not dexamethasone affects the severity and onset day of MVAC-induced severe neutropenia.

**Methods:**

This was a retrospective study of bladder cancer patients treated with MVAC chemotherapy with or without dexamethasone as an antiemetic at Kanazawa University Hospital during January 2005 - December 2009. Patients were categorized into three groups; no dexamethasone use (Dex (−)), dexamethasone on day 2 (Dex 1 day), and dexamethasone on days 2, 3 and 4 (Dex multiday). We evaluated the incidence of grade 3/4 neutropenia and the day of onset of first severe neutropenic episode during the first course of MVAC chemotherapy. Logistic regression was used to investigate whether co-administration of dexamethasone was a risk factor for severe neutropenia.

**Results:**

Episodes of grade 3/4 neutropenia occurred in 3 out of 6 (50.0%), 11 out of 12 (91.7%) and 6 out of 6 (100%) patients in the Dex (−), Dex 1 day, and Dex multiday groups, respectively. The appearance day of first severe neutropenia in the Dex multiday group (13.2 ± 1.0) was significantly accelerated compared to the Dex (−) group (17.7 ± 2.1). Univariate logistic regression analysis revealed that dexamethasone is a risk factor for severe neutropenia (OR 17.0; 95%CI: 1.3–223.1).

**Conclusions:**

Co-administration of dexamethasone for anti-emesis brings forward the first appearance of neutropenia, and increases the severity of neutropenia, in bladder cancer patients receiving MVAC chemotherapy.

## Background

Cancer chemotherapy with methotrexate, vinblastine, adriamycin and cisplatin (MVAC) is considered a standard therapy for urothelial cancer throughout the United States, Europe, Canada and Japan [1]. The MVAC regimen was developed about 30 years ago for transitional cell carcinoma of urothelium [1]. In a multicenter phase 3 trial, MVAC was shown to provide a survival advantage over cisplatin alone (median survival, 12.5 months vs 8.2 months, respectively) in patients with metastatic urothelial carcinoma [2]. However, the toxicity of MVAC is significant, with about 3–4% incidence of drug-related deaths, 20–25% incidence of nadir sepsis, and 58% incidence of grade 3/4 myelosuppression [1, 3]. Therefore, patients on MVAC therapy require frequent blood tests to monitor adverse effects.

Dexamethasone is effective for the prevention of emetogenic chemotherapy-induced nausea and vomiting (CINV) [4]. Anti-emetic guidelines such as those of the Multinational Association of Supportive Care in Cancer, the European Society of Medical Oncology, the American Society of Clinical Oncology, and the National Comprehensive Cancer Network recommend dexamethasone for prevention of CINV in patients given anti-tumor agents with low, moderate or high emetic risk [5, 6]. It was reported that dexamethasone enhances the anti-emetic effects of 5-HT_3_ receptor antagonists on vomiting induced by anti-tumor agents in ferrets [7–9]. Moreover, preclinical studies indicate that corticosteroid treatment prior to cytotoxic chemotherapy markedly reduced liver and bone marrow toxicity [10–13]. However, the safety of corticosteroid co-administration in this context has not been established.

In Kanazawa University Hospital Department of Urology, dexamethasone was initially not used for anti-emesis, because of potential side-effects such as increased susceptibility to infection, diabetes and osteoporosis. But, since vomiting and nausea in urothelial patients on MVAC therapy could not be controlled without dexamethasone, co-administration of dexamethasone with MVAC therapy was started in 2006. However, we noted an increase in the incidence of severe neutropenia and an earlier onset of first severe neutropenia in patients after the introduction of dexamethasone.

We found that relatively little is known about the side effects of dexamethasone in patients receiving emetogenic cancer chemotherapy [14], and in particular, it is not known whether co-administration of dexamethasone with cancer chemotherapy influences the severity or onset day of neutropenia.

Therefore, the aim of this study was to examine whether dexamethasone used as an anti-emetic affects the severity and/or frequency of neutropenia in bladder cancer patients receiving MVAC chemotherapy.

## Methods

### Data sources and cohort

This retrospective cohort study was conducted via a search of the computerized medical records at Kanazawa University Hospital. The files contain details of hospital procedures as well as patient demographics, laboratory data, drugs administered, doses, and date of administration. The study cohort was defined as all patients with urothelial cancer who received the first course of MVAC therapy at Kanazawa University Hospital from January 1st, 2005, to December 31th, 2009. Exclusion criteria included patients receiving an aprepitant, or corticosteroids other than dexamethasone.

The procedure of MVAC chemotherapy is as follows. A dose of 30 mg/m^2^ methotrexate is administered by intravenous (IV) infusion on days 1, 15 and 22. A dose of 3.0 mg/m^2^ vinblastine is administered by IV infusion on days 2, 15 and 22. Doses of 30 mg/m^2^ adriamycin and 70 mg/m^2^ cisplatin are administered by IV infusion day 2. The cycle is repeated every 28 days. Urothelial patients on MVAC therapy were classified according to the duration of dexamethasone co-administration. The survey covered six patients who received no dexamethasone (Dex (−)), twelve patients who received dexamethasone (8 mg) once on day 2 of MVAC (Dex 1 day), and six patients who received multiple administrations of dexamethasone (day 2, 8 mg; day 3, 2 mg; day 4, 2 mg) (Dex multiday). Granulocyte-colony stimulating factor (G-CSF) was used only when the neutrophil count fell below 1000 cells/μL neutrophils, in accordance with Japanese health insurance treatment recommendations during this study period. 5-HT_3_ antagonist (granisetron hydrochloride 3 mg or ondansetron hydrochloride hydrate 4 mg) was used 2 times on day 2 for all patients as another anti-emesis drug. Ticlopidine hydrochloride, aspirin, pravastatin, rosuvastatin calcium, bezafibrate, nifedipine, benidipine hydrochloride, and allopurinol, used as concomitant medicines, have been reported to cause leucopenia as a side effect. Other concomitant medications are described in Table 1.

**Table 1:** Patient demographics

	Dex (−) (*n* = 6)	Dex 1 day (*n* = 12)	Dex multiday (*n* = 6)
Age (year)	67.3 (58–73)	67.0 (57–79)	69.5 (57–76)
Sex (male/female)	5/1	12/0	3/3
PS (0/1/2)	5/1/0	10/1/1	6/0/0
Body surface area (m2)	1.8 (1.5–2.0)	1.7 (1.5–1.8)	1.6 (1.4–1.8)
BMI (kg/m2)	22.9 (19.8–28.1)	21.2 (15.9–27.5)	24.7 (19.3–30.0)
Creatinine clearance (mL/min)	94.5 (62.3–125.1)	99.8 (55.6–143.7)	98.5 (65.9–143.8)
Total bilirubin (mg/dL)	0.53 (0.4–0.8)	0.63 (0.3–1.4)	0.72 (0.5–1.3)
AST (IU/L)	24.7 (12–31)	22.0 (10–32)	18.3 (15–21)
Concomitant medications			
Oral 5HT3 receptor antagonists (%)	83.3	33.3	100
Dopamine antagonists (%)	50	33.3	50
Drugs reported leucopenia (%)	50	41.7	33.3
Purgative medicine (%)	50	50	50

Data are mean values (range in parentheses)

*PS* performance status, *AST* aspartate transaminase, *5HT* serotonin, *BMI* body mass index

Laboratory values were monitored at the physician’s discretion, but blood counts were consistently taken around day 15 (when methotrexate and vinblastine were administered). There was no significant difference of blood test measurement times among the Dex (−), Dex 1 day and Dex multiday groups in the first course of MVAC therapy (11.3 ± 2.2, 11.4 ± 2.5, and 10.0 ± 2.3, respectively).

The effects of baseline laboratory data (neutrophil counts <2000 cells/μL, total bilirubin >0.5 mg/dL, aspartate aminotransferase (AST) >27 U/L, creatinine clearance <60 mL/min), age of the patient (>65 y), body mass index (BMI) <23 kg/m^2^ and male sex) were evaluated, for comparison with a retrospective study on neutropenia in lung cancer patients [15]. BMI was calculated as weight (kg)/height^2^ (m^2^).

Nadir neutrophil counts after MVAC chemotherapy were recorded as the major outcome. We defined severe neutropenia as grade 3 or 4 neutropenia according to the Common Terminology Criteria for Adverse Events, version 4.0.

This study was carried out in compliance with the requirements of the Ethics Committee at Kanazawa University (protocol No.1008). All analyses were performed using anonymized data.

### Statistical analysis

Descriptive statistics were used for patients’ baseline clinical characteristics and the presence of drug administration. Continuous data are expressed as median with interquartile range. Categorical data are presented as frequency (percentage). Univariate logistic regression analyses were performed to identify risk factors for severe neutropenia. A *p* value less than 0.05 was considered significant.

All statistical analysis was performed using the statistical software package SPSS version 19.0J (SPSS Japan Inc., Tokyo) and Microsoft Excel 2010 (Microsoft Co. Ltd., Tokyo).

## Results

### Patient characteristics

The demographic characteristics of the patients are summarized in Table 1. There were no significant differences in age, sex, performance status, body surface area, BMI, creatinine clearance, total bilirubin, AST and concomitant medications among the Dex (−), Dex 1 day and Dex multiday groups (Table 1).

### Severity of severe neutropenia

The incidence of severe neutropenia increased with increasing number of days of dexamethasone, and the Dex multiday group showed a significantly greater incidence of grade 4 neutropenia compared to the Dex (−) group (Table 2).

**Table 2:** Severity of neutropenia in patients on the first course of MVAC chemotherapy

Neutropenia	Dex (−) (*n* = 6)	Dex 1 day (*n* = 12)	Dex multiday (*n* = 6)
G3 (%)	50.0	41.7	0
*P* value		1.0	0.18
G4 (%)	0	50.0	100.0
*P* value		0.054	0.002
G3/4 (%)	50.0	91.7	100.0
*P* value		0.08	0.18

*P* values are for G3, G4 and G3/4 neutropenia with Dex (1 day or multiday) versus DEX (−), as determined by Fisher’s exact test

### Day of onset of first severe neutropenia

The day of onset of first severe neutropenia was defined as the first detection day of neutrophil count <1000 cells/μL after MVAC chemotherapy (started on day 1). Dexamethasone significantly accelerated the day of onset of first severe neutropenia compared to the Dex (−) group, and the Dex multiday group showed the earliest onset (Table 3).

**Table 3:** Day of onset of first severe neutropenia in patients on the first course of MVAC chemotherapy

Neutropenia	Dex (−) (*n* = 3)	Dex 1 day (*n* = 11)	Dex multiday (*n* = 6)
Day of onset of severe neutropenia	17.7 ± 2.1	15.1 ± 2.1	13.2 ± 1.0
*P* value		0.113	0.008

Data are mean ± SD. *P* values are for day of onset of severe neutropenia with Dex (1 day or multiday) versus Dex (−), as determined by ANOVA with post hoc Tukey’s test

### Usage of G-CSF

Dexamethasone treatment tended to be associated with increased incidence of G-CSF usage compared to the Dex (−) group (Table 4). In particular, in the Dex multiday group, all patients required G-CSF. Also, the day of onset of the first G-CSF use was significantly accelerated compared to the Dex (−) group.

**Table 4:** G-CSF usage at the first course of MVAC chemotherapy

	Dex (−) (*n* = 6)	Dex 1 day (*n* = 12)	Dex multiday (*n* = 6)
G-CSF use (%)	50	75	100
*P* value		0.34	0.18
Day of Onset of first G-CSF use	18.0 ± 2.0	15.1 ± 2.7	13.7 ± 1.5
*P* value		0.17	0.04

*P* values are for G-CSF use with Dex 1 day and multiday versus Dex (−), as determined by Fisher’s exact test

Onset day of G-CSF first use data are mean ± SD. *P* values are for onset day of G-CSF use with Dex (1 day or multiday) versus Dex (−), as determined by ANOVA with post hoc Tukey’s test

### Risk factors for severe neutropenia

Univariate logistic regression analysis showed that dexamethasone use was associated with severe neutropenia, with odds ratios of 17.0 (95% CI 1.3 to 223.1) (Table 5). Neutrophil counts <2000 cells/μL and aspartate aminotransferase (AST) >27 U/L could not be analyzed because appropriate patients were not present in all groups. Other factors showed no association with severe neutropenia.

**Table 5:** Risk factors associated with Grade 3/4 neutropenia among 24 patients undergoing MVAC therapy at the first course, univariate logistic regression analysis

Variable	Incidence of severe neutropenia (%)	OR (95% CI)	*P* value
Dexamethasone	(+) : 94.4	17.0 (1.3–223.1)	0.03
	(−) : 50.0		
Age (>65 y)	(+) : 88.2	3.0 (0.33–27.2)	0.33
	(−) : 71.4		
Female	Female : 75.0	0.5 (0.04–7.0)	0.63
	Man : 85.0		
PS (>0)	(+) : 66.7	0.3 (0.02–4.9)	0.42
	(−) : 85.7		
Total bilirubin (>0.5 mg/dL)	(+) : 90.9	3.0 (0.3–34.0)	0.38
	(−) : 76.9		
BMI (<23 kg/m2)	(+) : 84.6	1.2 (0.1–10.5)	0.86
	(−) : 81.8		

*OR* odds ratio, *PS* performance status, *BMI* body mass index, *CI* confidence interval

## Discussion

This is the first report to suggest that co-administration of dexamethasone for anti-emesis induces severe neutropenia in patients receiving MVAC chemotherapy. The severity of MVAC-induced neutropenia was increased dose-dependently by dexamethasone, and the first onset of severe neutropenia was brought forward. Because severe neutropenia is an indication for reduced dose of anti-cancer agents, co-administration of dexamethasone appears likely to diminish the effectiveness of MVAC therapy. In particular, first severe neutropenia appeared around day 13 in the DEX multiday group, so that these patients would not be eligible to receive administration of methotrexate and vinblastine on day 15 of MVAC chemotherapy.

Current clinical practice guidelines recommend the use of G-CSF for prevention of febrile neutropenia [16], but during this clinical study period, G-CSF was given only when the neutrophil count fell below 1000 cells/μL, in accordance with Japanese health insurance treatment recommendations at the time. Thus, we considered that G-CSF would have had no effect on the incidence of severe neutropenia during MVAC therapy with dexamethasone. Usage of G-CSF tended to be greater in groups that received Dex treatment.

Ageing, poor performance status, advanced disease, certain comorbidities, low baseline values of blood cell counts, low body surface area/BMI, treatment with myelosuppressive chemotherapies, and specific genetic polymorphisms are reported to correlate with the risk of developing febrile neutropenia [17]. Although the present study did not include patients with advanced disease, certain comorbidities and specific genetic polymorphisms, univariate logistic regression analysis indicated that ageing, poor performance status, and BMI were not related to the incidence of severe neutropenia, and dexamethasone was the only risk factor identified for onset of severe neutropenia. The main reason for this difference may be the difference of chemotherapy regimens. MVAC chemotherapy frequently induces severe neutropenia compared to other chemotherapies, and therefore patients require careful monitoring.

Some reports have indicated that pretreatment with corticosteroids can induce tolerance to anti-cancer agents in vitro, or alter the pharmacokinetics of anti-cancer agents [10, 11]. Corticosteroid was also reported to confer resistance to cisplatin-induced apoptosis in human cervical and lung carcinoma cells [18]. However, our patients received dexamethasone simultaneously with MVAC, and so these mechanisms may not be relevant here.

Possible mechanisms through which dexamethasone might accelerate severe neutropenia include induction of granulocytosis and rapid progenitor cycling, which could heighten bone marrow chemo-sensitivity. This could occur through various mechanisms, such as enhanced release of polymorphonuclear leukocytes from bone marrow, delayed apotosis, and entry of polymorphonuclear leukocytes into inflamed tissues [19]. This idea is supported by evidence that patients receiving G-CSF concurrently with chemotherapy have more profound neutropenia or depressed neutrophil recovery as compared with historical controls or patients not treated with G-CSF and chemotherapy simultaneously [20, 21]. Another possible mechanism is drug-drug interaction between dexamethasone and MVAC components. Increased hepatotoxicity of methotrexate has been reported during dexamethasone therapy in humans [22] and rats [23]. This may be due to an increase of drug concentration in liver arising from reduced biliary elimination of the drug in the presence of dexamethasone [24]. However, it is not yet known whether or not the pharmacokinetics of vinblastine, cisplatin and adriamycin are affected by co-administration with dexamethasone in human and animals.

Our study has several limitations. 1) Since the data were obtained from clinical records, blood counts were not monitored as frequently as would be needed for accurate determination of nadir neutrophils, and the timing of blood counts was not the same for all subjects. 2) We could not determine the possible contribution to febrile neutropenia of other risk factors, including genetic polymorphisms, advanced disease, and certain comorbidities, although we did find that concomitant usage of other drugs had no apparent effect on neutropenia. Also, 3) our study is a single-center retrospective cohort study with relatively few subjects, so it is difficult to eliminate various possible biases and confounding factors. We could not add more patients to this clinical study, since dexamethasone should administered to patients treated with highly emetic chemotherapy according to current guidelines. Further research will be needed to establish the optimum dose and schedule of dexamethasone in individual patients, and to clarify the mechanisms through which dexamethasone affects hemocytes, including blood stem cells.

## Conclusions

We present the first evidence suggesting that co-administration of dexamethasone for anti-emesis increases the severity of neutropenia and brings forward the onset day of neutropenia in bladder cancer patients receiving MVAC chemotherapy.

## Abbreviations

AST: Aspartate aminotransferase
CINV: Chemotherapy-induced nausea and vomiting
CSF: Colony stimulating factor
Dex: Dexamethasone
IV: Intravenous
MVAC: Methotrexate, vinblastine, adriamycin and cisplatin
